# The effectiveness of pulsed electrical stimulation (E-PES) in the management of osteoarthritis of the knee: a protocol for a randomised controlled trial

**DOI:** 10.1186/1471-2474-9-18

**Published:** 2008-02-04

**Authors:** Robyn E Fary, Graeme J Carroll, Tom G Briffa, Ritu Gupta, N Kathryn Briffa

**Affiliations:** 1 Curtin University of Technology, School of Physiotherapy, Kent Street, Bentley, WA, 6102, Australia; 2 Arthrocare, 19A Guildford Road, Mount Lawley, WA, 6050, Australia; 3 University of Western Australia, School of Population Health, M431, 35 Stirling Hwy, Crawley, WA, 6009, Australia

## Abstract

**Background:**

Osteoarthritis (OA) of the knee is one of the main causes of musculoskeletal disability in the western world. Current available management options provide symptomatic relief (exercise and self-management, medication and surgery) but do not, in general, address the disease process itself. Moreover, adverse effects and complications with some of these interventions (medication and surgery) and the presence of co-morbidities commonly restrict their use. There is clearly a need to investigate treatments that are more widely applicable for symptom management and which may also directly address the disease process itself.

In two randomised controlled trials of four and 12 weeks duration, pulsed electrical stimulation was shown to be effective in managing the symptoms of OA of the knee. Laboratory and animal studies demonstrate the capacity of externally applied electric and electromagnetic fields to positively affect chondrocyte proliferation and extracellular matrix protein production. This latter evidence provides strong theoretical support for the use of electrical stimulation to maintain and repair cartilage in the clinical setting and highlights its potential as a disease-modifying modality.

**Methods/Design:**

A double-blind, randomised, placebo-controlled, repeated measures trial to examine the effectiveness of pulsed electrical stimulation in providing symptomatic relief for people with OA of the knee over 26 weeks.

Seventy people will be recruited and information regarding age, gender, body mass index and medication use will be recorded. The population will be stratified for age, gender and baseline pain levels.

Outcome measures will include pain (100 mm VAS and WOMAC 3.1), function (WOMAC 3.1), stiffness (WOMAC 3.1), patient global assessment (100 mm VAS) and quality of life (SF-36). These outcomes will be measured at baseline, four, 16 and 26 weeks. Activity levels will be measured at baseline and 16 weeks using accelerometers and the Human Activity Profile questionnaire. A patient global perceived effect scale (11-point Likert) will be completed at 16 and 26 weeks.

**Discussion:**

This paper describes the protocol for a randomised, double-blind, placebo-controlled trial that will contribute to the evidence regarding the use of sub-sensory pulsed electrical stimulation in the management of OA of the knee.

**Trial registration:**

Australian Clinical Trials Registry ACTRN12607000492459.

## Background

Osteoarthritis is a major cause of pain and disability in the community and OA of the knee is one of the most common causes of musculoskeletal disability in the Western world [1]. As prevalence increases it is expected to pose an increased burden on health care in the future.

Management options such as medication, exercise, self-management programs and surgery largely focus on providing symptom relief and maintenance of function, but do not, in general, address the disease process itself. Moreover, adverse effects and complications with some of these interventions (medication and surgery) and the presence of co-morbidities commonly restrict their use.

In recent years considerable effort has been directed towards investigating the effectiveness of putative disease-modifying OA drugs such as glucosamine, chondroitin sulfate, doxycycline and diacerein [2-5]. There is also interest in the use of pulsed electrical stimulation and electro-magnetic fields as potential OA disease modifying modalities. Laboratory work and animal studies provide theoretical support for the use of electrical stimulation to maintain and repair articular cartilage in the clinical setting [6-10]. However, there are limited studies examining the effects of pulsed electrical stimulation in humans.

Two randomised, placebo-controlled trials have reported using capacitively coupled pulsed electrical stimulation (PES) delivered via skin surface electrodes [11,12]. In both trials, outcome measures focussing on symptom relief and functional capacity have been the variables of interest.

The first of these trials [12] randomised 78 subjects to placebo or PES treatment with a monophasic, spiked signal at 100 Hz delivered by the Bionicare^® ^BIO-1000™. PES was applied for between six and 10 hours per day at an intensity just below the sensory threshold for four weeks.

Response to intervention was better for the active device than the placebo for the outcome measures of pain, physical function, physician global assessment and duration of joint stiffness in the morning (p < 0.05) [12]. No statistically significant difference was observed for range of knee joint motion, joint tenderness, joint swelling, knee circumference and 50 feet walking time.

The second PES trial [11] examined 58 subjects using the same stimulation device in the same manner over 12 weeks. In this trial significant and clinically meaningful results were reported in patient global assessment, a pain and symptom visual analogue scale, WOMAC function and stiffness and overall WOMAC score. Only WOMAC pain change between the placebo and active groups did not reach statistical significance.

No randomised, controlled trials studying this particular modality over a longer time period have been found.

The modality being investigated is neither invasive nor pharmaceutical. As the majority of those with OA of the knee are likely to be elderly, they are also more likely to have co-morbidities such as heart and lung disease which increase the anaesthetic risk associated with invasive surgery. Similarly, there is increasing awareness of the adverse side effects of many of the medications that are used to manage OA of the knee [13-15]. Consequently, in a climate where patients are seeking different options in their disease management strategies, the potential for PES to provide an effective, safe alternative that is acceptable to the community is very high.

This research proposes to investigate the longer term effectiveness of PES and to determine the sustainability of responses in subjects with symptomatic knee OA.

## Methods/Design

### Study design

**Figure 1:**
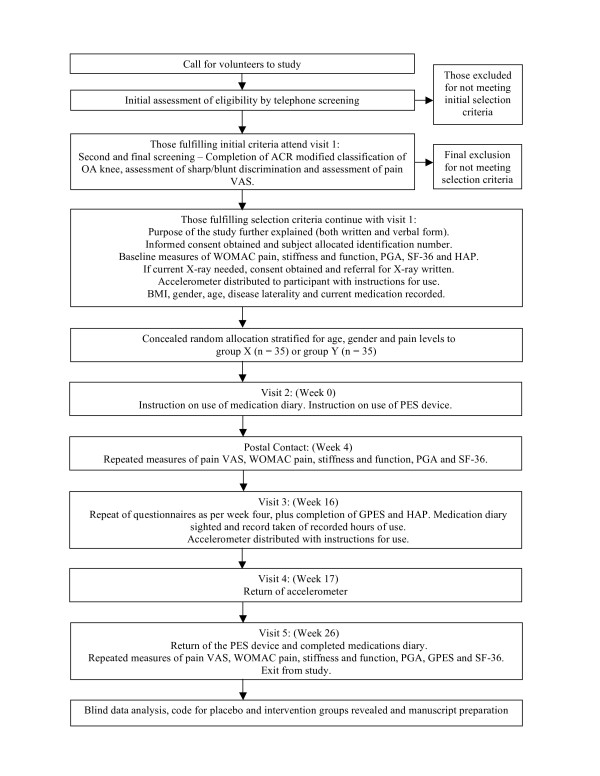
**Summary of study procedure**. ACR – American College of Rheumatology; WOMAC – Western Ontario and McMaster Universities Osteoarthritis Index; PGA – Patient Global Assessment; SF-36 – Medical Outcomes Study 36-item Short-Form Survey; HAP – Human Activity Profile; GPES – Global Perceived Effectiveness Scale.

A double-blind, randomised, placebo-controlled, repeated measure trial will be conducted over 26 weeks. Participants will be assessed, prior to the commencement of treatment (baseline), and after four, 16 and 26 weeks of treatment (Figure 1).

### Aims and hypothesis

The primary aim is to investigate for a period of 26 weeks, the effectiveness of transcutaneous PES in the treatment of symptomatic knee OA. A secondary aim is to determine whether effects, if any, are influenced by gender, age and/or baseline pain levels.

The experimental hypothesis is that PES will produce a clinically important and sustained improvement in pain, function, patient global assessment, global perceived effectiveness, quality of life and activity levels when compared with placebo treatment in individuals with symptomatic OA of the knee.

### Participants

Seventy participants with primary OA of the knee will be recruited.

### Inclusion criteria

• Primary knee OA diagnosed in accordance with the American College of Rheumatology (ACR) modified clinical classification criteria (sensitivity 84%, specificity 89%) [16,17]. This classification system has been shown to be a valid tool for OA knee diagnosis [18].

• Persistent, stable pain for a minimum of three months.

• Pain score of at least 25 mm on a 100 mm visual analogue scale (VAS).

### Exclusion Criteria

• Co-existing inflammatory arthropathies.

• Contraindications to electrical stimulation (pregnancy, decreased sensory perception, presence of metal in the field of application, or any implanted electrical stimulation device).

• Skin disorders in the treated knee area.

• Scheduled to have a total knee replacement within six months of entering the trial.

• Not able to read or understand English.

### Procedure

#### Recruitment

Potential volunteers will be recruited through community-based rheumatology and general practices, rheumatology outpatient clinics in teaching hospitals in the Perth metropolitan area, the Arthritis Foundation of Western Australia and promotion through media outlets.

#### Determining eligibility and baseline assessment

Initially volunteers will be telephone-screened to check for obvious exclusion criteria.

At a screening visit, diagnosis according to the ACR modified clinical classification will be made, sharp/blunt sensory discrimination will be tested and the pain VAS completed. Eligible participants who present with bilateral OA of the knee will be asked to nominate which knee they consider to be the most symptomatic and that knee will be treated.

Subjects who meet eligibility criteria will receive further information concerning the trial. In particular, the aims and methods will be explained in detail following which, written consent to participate will be sought. This trial has been approved by the Curtin University Human Research Ethics Committee (HR122/2006). Following consent, participants will be assigned an identification number and will be asked to complete baseline measures of WOMAC pain, stiffness and function, patient global assessment, quality of life and the Human Activity Profile (HAP) test, while details regarding body mass index (BMI), age, gender, laterality of joint disease, disease severity (if X-ray available) and current medication will be recorded. Participants who have not had a plain X-ray within the past two years will be referred for X-ray. Participants who are unwilling to have X-rays taken will not be excluded from the trial. Available X-rays will be graded according to the Kellgren and Lawrence radiological grading system. All outcome measurements will be taken and all instructions provided by an experienced musculoskeletal physiotherapist at Curtin University of Technology. At this first visit participants will also be provided with an accelerometer to collect seven days of ambulatory activity, after which it will be returned in person.

In the interim, participants will be randomly allocated into groups. Upon returning the accelerometer, they will be fitted with the PES device and given detailed verbal and written instructions regarding its use. They will also be provided a medications diary and relevant instructions.

### Randomisation and blinding

**Figure 2:**
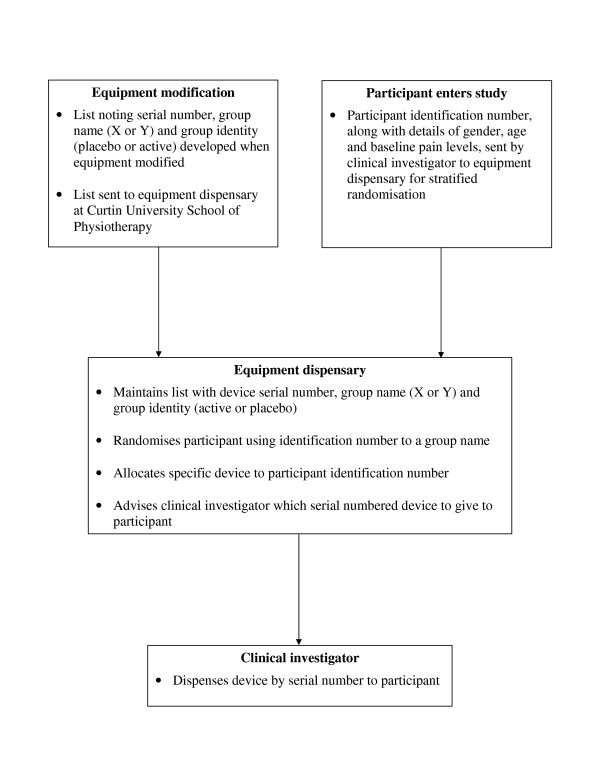
Process for stratified randomisation and concealed allocation.

An administrator, not otherwise involved with the trial, will allocate the participants to groups using computer-generated block randomisation combined with stratification. Groups will be stratified with regard to gender, age (<60, 60–75 and >75), and intensity of pain (VAS scores 25–40, 41–60 and 61–100). The administrator will dispense an appropriate device using a list, provided by the senior biomedical engineer who modified the devices, that matches the device serial numbers to active or placebo. Participant identification will be added to the list at the time of randomisation so, should the need arise, the investigators will be able to determine the nature of the device provided to a participant without risk of becoming aware of assignment for any other participants (Figure 2). The measurer though will remain blinded to assignment throughout.

The group-to-device-to-intervention code will remain in the possession of the equipment dispensary (protected by password) until after the data analysis has been completed.

### Trial intervention

A commercially available transcutaneous electrical nerve stimulator (Metron Digi-10s) has been adapted by a senior biomedical engineer to produce either a pulsed, exponentially declining waveform with a frequency of 100 Hz or a placebo device identical in appearance. Participants will be advised to use the device at a sub-sensory level, to replicate the conditions in the previous randomised controlled trials, for a minimum of seven hours per day. It will be recommended that they wear the device overnight. There is a locking mechanism on the device so that once the treatment intensity has been set there can be no inadvertent intensity change during the course of treatment. The instructions given to participants by the physiotherapist, who remains blind to the nature of the device, will be exactly the same regardless of which device participants are using.

### Background therapy

Participants will be asked to continue their normal background medical management during the trial period. A medications diary will be used to alert the investigators to any changes in medication usage. Each subject's managing doctor will be informed by letter of their patient's participation in the trial. Doctors will be requested to refrain from changing OA management during the period of the trial if at all possible.

### Compliance

Participants will be asked to keep a record of their daily PES use. This will be maintained within the medications diary booklet. Intermittent questioning about PES use via regular phone calls during the trial period and a review of the booklet at 16 weeks will occur.

### Safety monitoring and adverse events

Participants will be encouraged to contact the investigators should any questions arise during the trial. Correspondingly, they will be asked open-ended questions by the physiotherapist during the scheduled phoned calls to determine any adverse effects from using the device. The only adverse reaction that is expected is mild skin irritation. This occurred in both the placebo and intervention groups in both randomised controlled trials in approximately equal proportions (range 17.9–24% active and 21 – 21.1% placebo) and responded favourably to topical therapy, a temporary halt in use and/or change in the conduction gel [11,12]. Any participant reporting skin irritation will be asked to stop using the device and to return to the Curtin University School of Physiotherapy for assessment at the first available opportunity. Should there be pain, swelling or more than a five centimetre diameter area of redness after desisting for 48 hours, a medical opinion will be obtained before proceeding further.

### Outcome measures

Outcome measures include the core set of primary efficacy variables recommended by the Outcome Measures in Rheumatology Clinical Trials (OMERACT) group for phase III clinical trials in OA [19] and recommended for inclusion in OA clinical trials by the Cochrane Collaboration [20].

The three *primary efficacy variables*, to be measured at baseline, four, 16 and 26 weeks, are:

• Pain (100 mm VAS and Western Ontario and McMaster Universities Osteoarthritis Index -WOMAC Likert format 3.1). Two measures for pain will be included as this mirrors standard practice in much of the OA literature and it will provide internal validity for this important outcome measure. The reliability of the VAS has previously been demonstrated [21] and is regularly used in this population. WOMAC measures health status and assesses pain, physical function and stiffness in patients with OA of the hip or knee. The WOMAC questionnaire is self-administered and can be completed in less than five minutes. Two major validity studies have shown that WOMAC pain, physical function and stiffness subscales are valid and that the questionnaire is reliable and sensitive enough to detect changes in health status following a variety of interventions [22].

• Patient Global Assessment (100 mm VAS as described by Ehrich et al [23]). Participants are asked to consider all the ways in which their arthritis is affecting them at the time of the assessment and indicate by marking the VAS how they are doing. The left hand anchor of the VAS is *Very Well *while the right hand anchor is *Very Poorly*.

• Physical function (WOMAC Likert format 3.1). Likert values from 17 questions of the WOMAC are summed to generate a score for physical function with a higher score indicating worse function.

#### Secondary outcome measures

• Quality of life (Medical Outcomes Study 36-item Short-Form Survey version 2 – SF-36). The SF-36 has eight sub-component scales reflecting both physical and mental status. It is comprised of 36 questions, is self administered, and can be completed in about 15 minutes. All estimates of score reliability, from 14 separate studies, for each of the eight scales of the SF-36 exceeded accepted standards for measures used in group comparisons [24]. The SF-36 has been extensively validated in many English speaking countries of the world including Australia [25]. The rationale for using both WOMAC and the SF-36 for this trial is that a combined approach using both generic quality of life and knee specific health status measures is considered likely to prove best for knee-related problems [26]. Quality of life measurements will be taken at baseline, four, 16 and 26 weeks.

• Joint stiffness (WOMAC Likert format 3.1) measured at baseline, four, 16 and 26 weeks.

• Global perceived effect scale (GPES) measured using an 11-point scale ranging from -5 (vastly worse) to +5 (completely recovered) with the zero point being unchanged as reported by Pengal et al [27]. This will be measured at 16 and 26 weeks.

• Physical activity will be determined using the HAP questionnaire plus accelerometers measured at baseline and 16 weeks.

◦ The HAP is self-administered. It incorporates 94 activities listed in ascending order of oxygen cost. Respondents are asked to indicate whether they are able to perform the activity unassisted; whether they have ever performed the activity and whether they have stopped performing the activity [28]. In a cross-sectional study, the HAP has been found reliable and sufficiently sensitive for use in people with OA of the knee [29]. This study demonstrated that people with OA of the knee are in fact less active than their healthy counterparts and that there was a relationship between HAP scores and participants' pain and function scores. The proposed trial offers the potential to determine whether improvement in pain and function result in a spontaneous increase in the level of physical activity.

◦ Accelerometry is now considered the preferred method of objectively measuring physical activity as it provides data that allows individual examination of ambulatory activity frequency, intensity and duration [30]. However, to our knowledge accelerometers have not been validated for use in people with OA of the knee. For this trial an Actigraph GT1M (formerly Computer Science and Applications monitor), the most widely accepted accelerometer in research, will be utilised [31]. The match-box size accelerometer will be attached to a belt that the participants will be asked to wear for a period of seven consecutive days at both the baseline and 16 week data collection points.

The benefits of physical activity are well known and include reduced risk of cardiovascular disease, diabetes, some forms of cancer, osteoporosis, falls and fractures. Physical activity interventions have also been shown to assist with weight control and to improve physical functioning and mental health. The impact of physical activity on OA remains unanswered. Osteoarthritis of the knee is often associated with considerable knee pain that suggests that people with knee OA may curtail their physical activity. Although most studies of OA of the knee measure changes in function, few measure physical activity levels so there is limited evidence to demonstrate whether or not this is the case. Moreover whether treatments that reduce pain result in an increase in physical activity is yet to be determined.

### Sample Size Calculations

The primary outcome measure will be defined as an improvement in the absolute pain VAS score of 20. This is the minimum absolute change necessary for classification as a responder in the Osteoarthritis Research Society International (OARSI) response criteria [32,33]. Assuming no change in the placebo group, it has been calculated that a sample size of 70 (35 in each group allowing for 20% withdrawals) will be sufficient to detect this 20 point change as well as differences equal to the absolute minimal clinically important improvements of 19.9 (Pain VAS), 18.3 (PGA) and 9.1 (WOMAC function) described by Tubach et al [34] with a power of 80% using a two-tailed test with alpha level of 0.05. Calculations were based on standard deviations data from Garland et al [11] (Pain VAS and PGA) and Raynauld et al [35] (WOMAC function).

### Statistical Analysis

All analyses will be performed on an intention to treat basis while the investigators remain blind to treatment groups. Change in pain between baseline and 26 weeks will be compared between groups using the independent t-test. To test for the fixed effect of treatment while adjusting for any differences in the baseline measures, repeated measures analysis using a linear mixed model will also be performed for pain VAS, patient global assessment, WOMAC function, stiffness and pain, and QOL measurements taken at four, 16 and 26 weeks. There will be no adjustment for multiple comparisons as all comparisons have been determined a priori and, while adjustment maintains study wise error, it may preclude detection of clinically important differences [36].

Change in activity level between baseline and 16 weeks will also be analysed using the independent t-test. Secondary analyses such as the proportion of participants achieving minimal clinically important improvements in pain, function and patient global assessment at each observation time will be compared using the Chi-square test. GPES at 16 weeks and 26 weeks will be compared between groups using unpaired t-tests.

### Ethical considerations

A placebo control is being used in this trial. However, as subjects are not being asked to change their usual treatment regimen, no subject will be disadvantaged by using the placebo.

### Data Quality

Data will be entered into a specifically designed database with pop-up value lists, value ranges, data type and field complete validations. Random scrutiny by co-investigators of at least ten percent of all data entered will be conducted throughout the trial to ensure accuracy and completeness.

### Timelines for E-PES trial

Patient recruitment and initial phone screening began in July 2007. Final screening and data collection commenced in October 2007 with final exit data expected to be collected in February 2009.

## Discussion

This paper describes the rationale and protocol for conducting a double-blind, randomised, placebo-controlled trial that will investigate the use of pulsed electrical stimulation in the management of OA of the knee. It incorporates features designed to minimise bias [37] and uses valid outcome measures that will facilitate comparability with other research in the area.

This trial will contribute to the evidence regarding the use of a non-pharmaceutical, non-invasive modality in managing symptoms of OA of the knee. Given the modality's simple technology and ease of use (patients can readily use it at home), it has huge potential to provide a safe, effective treatment option for clinicians.

## Competing interests

The author(s) declare that they have no competing interests.

## Authors' contributions

All authors were responsible for identifying the research question and contributing to drafting the trial protocol. Robyn Fary has been responsible for the drafting of this paper, although all authors have provided substantial input, providing comments on the drafts and have read and approved the final version.

## Pre-publication history

The pre-publication history for this paper can be accessed here:


